# Exploring Abnormal Brain Functional Connectivity in Healthy Adults, Depressive Disorder, and Generalized Anxiety Disorder through EEG Signals: A Machine Learning Approach for Triple Classification

**DOI:** 10.3390/brainsci14030245

**Published:** 2024-03-01

**Authors:** Jiaqi Fang, Gang Li, Wanxiu Xu, Wei Liu, Guibin Chen, Yixia Zhu, Youdong Luo, Xiaodong Luo, Bin Zhou

**Affiliations:** 1 College of Engineering, Zhejiang Normal University, Jinhua 321004, China; fjq0971@zjnu.edu.cn (J.F.); xwx@zjnu.edu.cn (W.X.); lyd@zjnu.cn (Y.L.); 2 College of Mathematical Medicine, Zhejiang Normal University, Jinhua 321004, China; ligang@zjnu.cn; 3 College of Computer Science and Technology, Zhejiang Normal University, Jinhua 321004, China; liuwei@zjnu.edu.cn (W.L.); cgbcvip@zjnu.edu.cn (G.C.); 4 The Second Hospital of Jinhua, Jinhua 321016, China; zyxjhey@163.com

**Keywords:** depression disorder, generalized anxiety disorder, electroencephalogram (EEG), functional connectivity, machine learning

## Abstract

Depressive disorder (DD) and generalized anxiety disorder (GAD), two prominent mental health conditions, are commonly diagnosed using subjective methods such as scales and interviews. Previous research indicated that machine learning (ML) can enhance our understanding of their underlying mechanisms. This study seeks to investigate the mechanisms of DD, GAD, and healthy controls (HC) while constructing a diagnostic framework for triple classifications. Specifically, the experiment involved collecting electroencephalogram (EEG) signals from 42 DD patients, 45 GAD patients, and 38 HC adults. The Phase Lag Index (PLI) was employed to quantify brain functional connectivity and analyze differences in functional connectivity among three groups. This study also explored the impact of time window feature computations on classification performance, including the XGBoost, CatBoost, LightGBM, and ensemble models. In order to enhance classification performance, a feature optimization algorithm based on Autogluon-Tabular was proposed. The results indicate that a 12 s time window provides optimal classification performance for the three groups, achieving the highest accuracy of 97.33% with the ensemble model. The analysis further reveals a significant reorganization of the brain, with the most pronounced changes observed in the frontal lobe and beta rhythm. These findings support the hypothesis of abnormal brain functional connectivity in DD and GAD, contributing valuable insights into the neural mechanisms underlying DD and GAD.

## 1. Introduction

Mental illnesses, particularly depressive disorder (DD) and generalized anxiety disorders (GAD), have garnered widespread attention across various sectors due to their escalating prevalence year after year. DD is mainly characterized by a significant and persistent depressed mood, and some patients have self-injurious and suicidal behaviors [1]. GAD is a subtype of anxiety disorder (AD), characterized by unconscious, persistent tension and anxiety, accompanied by obvious fidgety somatic manifestations [2]. By 2020, there were 193 million people living with DD and 374 million with AD worldwide [3]. Among these figures, over 58 million were estimated to be children and adolescents [4,5]. The clinical diagnosis of mental disorders is standardized by The Diagnostic and Statistical Manual of Mental Disorders IV (DSM-IV) [6], which clearly states that DD and GAD are separate entities. However, discerning between DD and GAD remains challenging due to their similarity in clinical symptoms [7,8]. Physicians often rely on a combination of self-assessment scales (such as the Hamilton Anxiety Scale, Depression Scale, Beck Depression Inventory, etc.) and clinical experience for patient diagnosis, which involves a significant subjective element. Therefore, there is an urgent need for an objective diagnostic approach to provide scientifically grounded diagnostic criteria.

An electroencephalogram (EEG), a noninvasive approach for capturing bio-electrophysiological signals, is increasingly applied in both research and clinical contexts related to psychiatric disorders such as DD and GAD. This predilection is ascribed to its notable advantages, including high temporal resolution, relatively affordable implementation, and safety, with no associated radiation risk [9,10]. In EEG signals, there are often numerous artifacts. In previous studies [11,12,13], we utilized an independent component analysis (ICA) combined with manual artifact removal to achieve satisfactory results. Additionally, traditional methods of EEG analysis involve linear analysis [14], nonlinear analysis [15], and brain functional connectivity analysis [16]. Among these, the Phase Lag Index (PLI) represents a prevalent method for analyzing brain functional connectivity, and its strength lies in its minimal sensitivity to volume conduction effects. It has been confirmed as an effective method for distinguishing mental disorders such as DD and GAD through the calculation of EEG features [11,12,17]. The research on Qi reveals that PLI, in comparison to linear and nonlinear features, exhibits superior classification performance [13]. Moreover, the frequency band activities in EEG serve as reflective indicators of the subject’s mental state [18]. For instance, aberrant activity in the beta band is often associated with states of anxiety and depression [19]. Guo et al.’s study highlights the increased abnormality in long-range functional connections in the beta band within the frontal region of DD patients, manifested as asymmetry in the frontal lobe structures [20]. Additionally, research on anxiety disorders similarly reveals a significant reorganization of brain functional connectivity in the frontal region within the beta band [5,21,22]. Patients diagnosed with DD and GAD exhibit significantly different patterns of brain reorganization, highlighting notable individual variability in their neural responses. The validation of these observed patterns requires extensive research experiments, as they play a crucial role in investigating the underlying mechanisms.

Machine learning (ML) is frequently employed in conjunction with EEG features for the intelligent classification of mental illnesses [23,24,25]. Ensemble learning, a type of ML, achieves enhanced learning performance by constructing multiple learners. Notably, three gradient boosting ensemble methods, light gradient boosting machine (LightGBM) [26], extreme gradient boosting (XGBoost) [27], and categorical boosting (CatBoost) [28], have demonstrated efficiency and accuracy as classifiers in supervised ML tasks [29]. A recent study showed that XGBoost demonstrated superior classification performance with an accuracy of 99% for both DD and GAD groups, outperforming LightGBM (98%) and RF (95%) under identical experimental conditions [9]. Sau and Bhakta trained RF (89%), which had a better prediction than random tree (85%) [24]. Similar studies achieved 97% [21], 89% [23], and 82.6% [24] accuracy in categorizing AD and HC groups, respectively. Different studies reported using completely different experimental data, and it is difficult to evaluate their results. However, the results of all these studies point out that ML is feasible and effective for the diagnosis of DD, GAD, and HC.

This study innovatively proposes a data-driven diagnostic approach for understanding the mechanisms of GAD and DD. Specifically, we utilize PLI features and introduce an innovative ensemble feature optimization algorithm based on Autogluon-Tabular. By analyzing the optimal feature subset, we build brain functional networks to examine the differences in brain functionality between GAD, DD, and HC. Additionally, this study revealing the impact of computing features over different time window lengths on classification performance. We anticipate enhancing the model’s classification accuracy while elucidating potential underlying mechanisms.

## 2. Materials and Methods

### 2.1. Participants and Materials

EEG data were obtained from Huzhou Third Hospital, including 38 individuals in good health, 45 individuals with GAD, and 42 individuals with DD. All participants were clinically diagnosed by physicians before data collection, meeting the clinical criteria for AD and DD as the DSM-IV. They were also assessed using the Hamilton Anxiety Scale (HAMA) and Hamilton Depression Scale (HAMD) [30]. Criteria for inclusion involved GAD participants having HAMA > 14, HAMD < 7, and DD participants having HAMA < 7, HAMD > 17. Importantly, there was no comorbidity between the two groups. Through a statistical analysis, it was ensured that gender and age were independent, as shown in Table 1. According to statistics, the GAD group has an average HAMA value of 25.09 ± 9.00, and the DD group has an average HAMD value of 24.95 ± 7.06.

Additionally, participants were required to meet specific criteria, including right-handedness, the absence of other mental disorders (such as epilepsy or bipolar disorder), and no physical brain abnormalities. To prepare for the data collection experiment, participants were instructed to ensure that they had adequate sleep and abstained from alcohol, smoking, and high-caffeine beverages on the preceding day.

### 2.2. EEG Data Acquisition and Preprocessing

**Figure 1:**
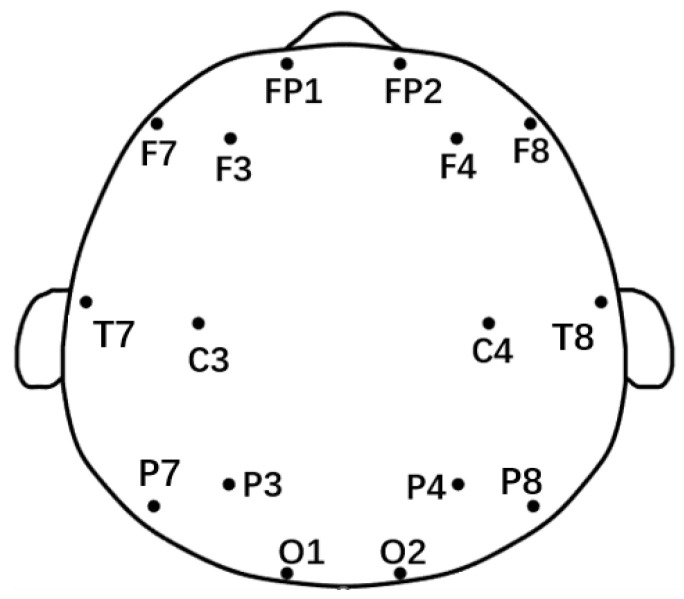
The 10–20 electrode distribution and segmentation according to brain regions.

Nicolet EEG TS215605 equipment (Nicolet Instruments, Madison, WI, USA) was utilized for this experiment, which took place in a peaceful and warm environment. Participants were instructed to keep their eyes closed, maintain a comfortable posture, and avoid falling asleep. the equipment captured EEG data from 16 electrodes, as shown in Figure 1, with the left and right earlobes serving as reference points. This setup allowed the simultaneous collection of both horizontal and vertical ophthalmoplegia to differentiate ocular artifacts. The sampling frequency of the EEG signal was 250 HZ, and the impedance value of each electrode was guaranteed to be less than 5 KΩ during the experiment. The experiment collected 10 min of EEG data from each participant.

An EEG data preprocessing experiment was designed to enhance data quality by eliminating artifacts, including ocular and muscle-related interference. The pre-processing primarily involved band-pass filtering (4–30 Hz) to remove noise interference, baseline drift correction, down-sampling to 125 Hz to reduce computational load, and the application of ICA to remove artifact components. To explore the influence of feature computation with varying time window lengths on the results, the experiment employed sliding time windows ranging from 2 to 16 s, without any overlay. As shown in Table 2, the sample sizes for each group were determined based on the division of data into different time window lengths. Additionally, the experiment utilized band-pass filtering to extract four specific frequency bands of interest—Theta (4–8 Hz), Alpha1 (8–10 Hz), Alpha2 (10–13 Hz), and Beta (13–30 Hz).

### 2.3. Feature Extraction

The PLI was employed to calculate the degree of phase synchronization between time series signals, which is proven to be effective in characterizing changes in functional networks. In this study, it is utilized as a metric to describe functional connectivity, where larger PLI values indicate a higher degree of phase synchronization between signals from two channels.

Two-channel EEG time series signals are denoted as *S*_1_(*t*) and *S*_2_(*t*). The PLI calculation is as follows. First, its instantaneous phase is calculated, as shown in Formula (1). It transforms the time domain signal into a time–frequency domain signal. Next, the phase difference is calculated using Formula (2). This involves calculating the magnitude and phase of the complex signal, derived from the phase information at each time point. Finally, the PLI value is obtained by Formula (3).

(1) $$Z_{i}\left(t\right)=S_{i}\left(t\right)+jHT\left(S_{i}\left(t\right)\right)$$

(2) $$\Delta \varphi \left(t\right)=\arg\left(\frac{Z_{1}\left(t\right)*Z_{2}\left(t\right)}{\left|Z_{1}\left(t\right)\right|*\left|Z_{2}\left(t\right)\right|}\right)$$

(3) $$PLI=\left|\langle sign\left(\Delta \varphi \left(t\right)\right)\rangle \right|=\left|\frac{1}{N}\overset{N}{\underset{n=1}{\sum }}sign\left(\Delta \varphi \left(t\right)\right)\right|$$

In this context, $Z_{i}\left(t\right)$ represents the EEG time domain signal, $\Delta \varphi \left(t\right)$ denotes the phase difference between two sets of time domain signals, and sign refers to the sign function.

For each sample within a single frequency band, a total of 16 × (16 − 1)/2 = 120 *PLI* features were extracted. Since each sample consists of 4 frequency bands, this results in a total extraction of 4 × 120 = 480 *PLI* features per sample.

### 2.4. Machine Learning

The tree model based on a decision tree has been widely used in the field of disease detection and has shown excellent detection performance. In particular, the gradient boosting decision tree (GBDT), as a machine learning model, achieves the purpose of constructing an optimal model by iteratively training a weak classifier (decision tree). This method has many advantages, such as effective training and anti-overfitting. In this study, three improved models (LightGBM, XGBoost, and CatBoost) based on the GBDT model and an ensemble model were used.

**Figure 2:**
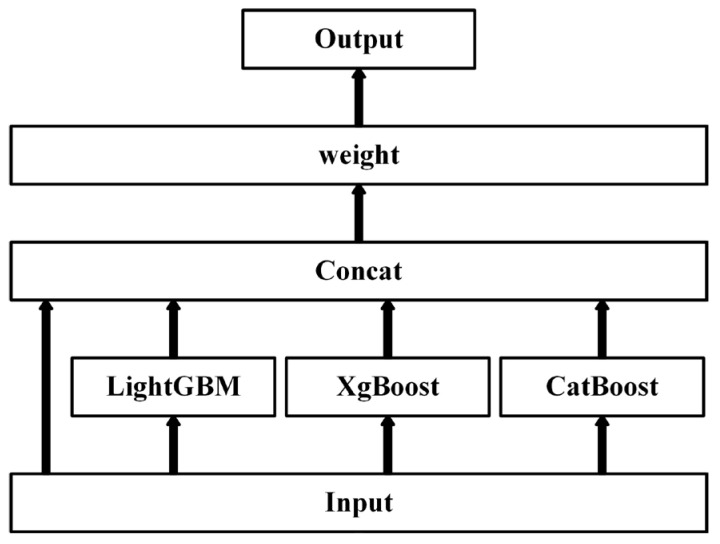
The feature optimization framework.

(1) LightGBM supports efficient parallel training, offering faster training speeds, lower memory consumption, improved accuracy, and the ability to handle massive data efficiently. It also supports distributed computing, making it suitable for processing large datasets. 

(2) XGBoost significantly improves its efficiency, accuracy, and robustness in predictive modeling by integrating parallel computation, optimizing the loss function using second-order derivatives, and introducing L1 and L2 regularization techniques.

(3) CatBoost addresses issues such as gradient bias and prediction shift, reducing the occurrence of overfitting and thereby enhancing the algorithm’s accuracy and generalization capability. It is also suitable for processing handling categorical features.

(4) Furthermore, a shallow stacked ensemble of the above three models is performed using Autogluon [31]. This ensemble model takes as inputs not only the predictions of the previous layer of models, but also the raw data features themselves (the input vectors are the data features connected to the predictions of the lower layer of models), and the final stacked layer applies the ensemble choice to aggregate the predictions of the stacked models in a weighted manner, as shown in Figure 2.

### 2.5. Feature Selection

Feature selection, also known as feature optimization, is a common approach for dimensionality reduction in ML. It involves a reduction in data dimensions to enhance model performance, generalization capabilities, and algorithm efficiency. This study innovatively proposes a feature selection framework suitable for ensemble learning, referred to as the Multi-Model Joint Feature Selection Algorithm. The purpose of this algorithm framework is to identify feature subsets that perform well across three base models (LightGBM, Catboost, and Xgboost), as well as the ensemble model, for subsequent mechanism analysis. The algorithm framework consists of two processes: feature ranking and subset selection.

The specific process is as follows. Firstly, feature ranking involves analyzing the importance of each feature using a specific algorithm, sorting them based on their contributions to the model performance. Specifically, *k*-fold cross-validation is used to partition the data set (where *k* − 1 fold is the training set, and 1 fold is the test set). Additionally, 10% of the training set was reserved as a validation set. For each base model, a classifier was trained using the training dataset, and model parameters were optimized using the validation set. Feature importance was then determined using the testing set data and the feature_importance method available in Python for tree model ensembles. Subsequently, based on the feature importance values, different feature weights were assigned, and all features were sorted to obtain a set of features ranked in descending order of importance. A higher weight indicates a greater importance of the feature in the model’s decision-making process. To enhance generalization ability, the experiment further employed repeated p times of *k*-fold cross-validation, and the descending feature matrices of the three base models were concatenated for subsequent subset selection. The calculation formula is represented as Formula (4):

(4) $$Index=concat\left(I_{LightGBM},I_{Catboost},I_{Xgboost}\right)$$

where $I_{LightGBM},\;I_{Catboost}$ and $I_{Xgboost}$ are the index results of multiple training and ranking iterations for LightGBM, Catboost and Xgboost. *Index* represents the concatenated results of the ranking outcomes from the three base models. $I_{LightGBM},\;I_{Catboost},\;I_{Xgboost}\in R^{\left(p*k\right)\times d_{feature}}$, $Index\in R^{\left(3*p*k\right)\times d_{feature}}$. Here, *p* is set to 100, *k* is 5, and $d_{feature}$ is the number of features (which is 480 in this study).

Next, subset selection aims to construct the optimal feature subset to extract key functional connectivity features for further mechanistic analysis. Based on the sorted feature ranking results, features are arranged in frequency order to capture the most frequently occurring features. These features are then selected as elements of the optimal feature subset. Specifically, for the feature ranking, *Index*, obtained from multiple trainings, we select the top n columns one by one, each containing the n most frequent features, to construct feature subsets. We trained all subsets using the data partitioning method in feature ranking (*p* set to 5, *k* set to 5) and obtained results, selecting the best-performing feature subset in the ensemble model as the optimal feature subset. This method aims to capture potentially stable and significantly predictive feature subsets by considering the relative importance of features across multiple training iterations and their frequent occurrence across the entire dataset.

### 2.6. Parameter Optimization

In the field of ML based on tree models, hyperparameters are critical factors influencing model performance and generalization ability. Different combinations of parameters can lead to vastly different training results, making the selection of hyperparameters crucial. Appropriately tuning hyperparameters can significantly reduce the risk of overfitting or underfitting, thereby enhancing the model’s predictive accuracy and robustness [32]. Therefore, in this study, we employ Bayesian optimization, an efficient method aimed at discovering the optimal hyperparameter combinations to optimize model performance. 

This process involves three pivotal components: defining the objective function, setting the hyperparameter search space, and specifying the number of search iterations. Firstly, we chose *F*1-macro as the optimization target, a comprehensive performance metric suitable for multi-class classification problems. Secondly, we referred to the Autogluon automatic ML framework [31] to set various critical hyperparameters search space. Lastly, we set the maximum number of searches to 30 to thoroughly explore hyperparameter combinations within a limited time. The parameter optimization ranges for LightGBM, XGBoost, and CatBoost are shown in Table 3.

### 2.7. Evaluating Indicator

In this study, we evaluate the performance of the model in a multi-classification task using a range of metrics, including accuracy, *F*1-macro, Gmean-macro, and kappa. Accuracy represents the proportion of correctly predicted samples out of the total samples. *F*1-macro is the macro-average of the *F*1 score, where the *F*1 score is the harmonic mean of precision and recall. Gmean-macro is the macro-average of the geometric mean, designed to comprehensively consider the classifier’s performance for each class. Kappa is a metric used to measure the consistency between a classifier and random classification. These metrics, with values ranging from 0 to 1, tend toward 1 as the model’s performance improves, and their computation processes are outlined below.

(5) $$\mathrm{Accuracy}=\frac{TP_{HC}+TP_{GAD}+TP_{DD}}{All\;samples}$$

where *TP* stand for true positives (correctly predicted positives). Here, $TP_{HC}$ represents the number of correctly predicted samples in the HC group, $TP_{GAD}$ represents the number of correctly predicted samples in the GAD group, and $TP_{DD}$ represents the number of correctly predicted samples in the DD group, and ‘*All samples*’ represents the total number of samples.

(6) $$F1-\mathrm{macro}=\frac{F1_{1}+F1_{2}+\cdots +F1_{N}}{N}$$

Among them, *N* is the number of categories, and $F1_{1}$, $F1_{2}$, … $F1_{N}$ represent the *F*1 score for each category.

(7) $$\mathrm{Gmean}-\mathrm{macro}=\sqrt{\frac{1}{N}\sum_{i=1}^{N}Precision_{i}\times {\mathrm{Recall}}_{i}}$$

Among them, *N* is the total number of categories, and $Precision_{i}$ represents the precision of the i-th category, ${\mathrm{Recall}}_{i}$ represents the recall rate of the i-th category.

(8) $$\mathrm{kappa}=\frac{P_{o}-P_{e}}{1-P_{e}}$$

Among them, $P_{o}$ is the observed consistency of classification, which is also the accuracy of classification. $P_{e}$ is the expected consistency of classification, and the specific calculation method is as follows:

(9) $$P_{e}=\frac{TP_{HC}+TP_{GAD}+TP_{DD}}{All\;samples^{2}}$$

## 3. Results

**Figure 3:**
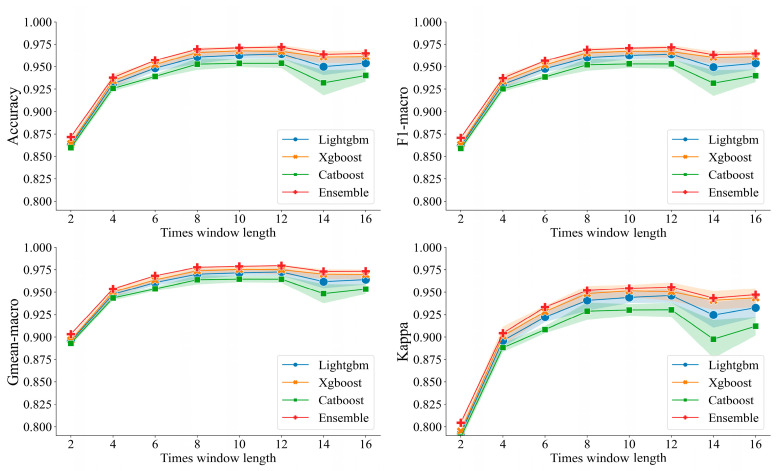
Analysis of classification performances across various models within segmented time windows.

Figure 3 shows the performance comparison of the LightGBM, XGBoost, CatBoost, and ensemble models across various scenarios of time window feature computation. We can see that as the length of the time window increases, the performance of the model shows an increasing trend and the model obtains the best performance at 12 s, as shown in Table 4. Among the models, the ensemble model obtained the best classification performance (accuracy of 96.89%, *F*1-macro score of 96.86%, Gmean-macro of 95.26%, and Kappa value of 97.65%.) compared with LightGBM, XGBoost, and CatBoost models. As a result, all subsequent analyses in this study were conducted based on feature computation using a 12 s time window.

**Figure 4:**
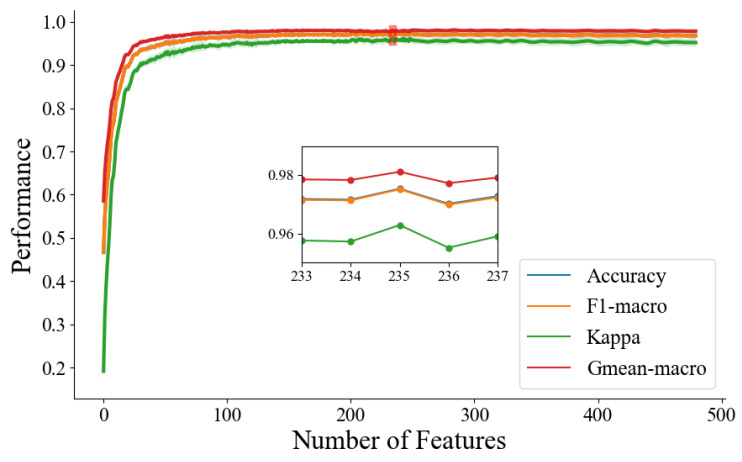
The iterative graph of the optimal feature subset in the feature selection process.

**Figure 5:**
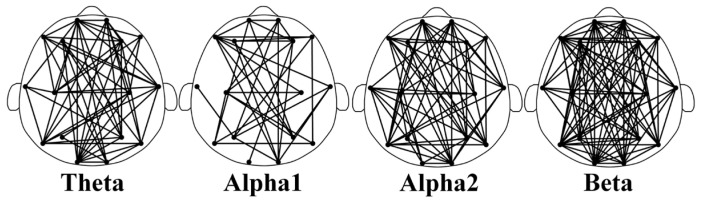
The distribution of differential key functional connections within the theta, alpha1, alpha2, and beta rhythms among the HC, GAD, and DD groups. These distinctions are derived from an optimal feature subset (235 edges in total), where the number of four rhythms is 54, 35, 61, and 85, respectively.

**Figure 6:**
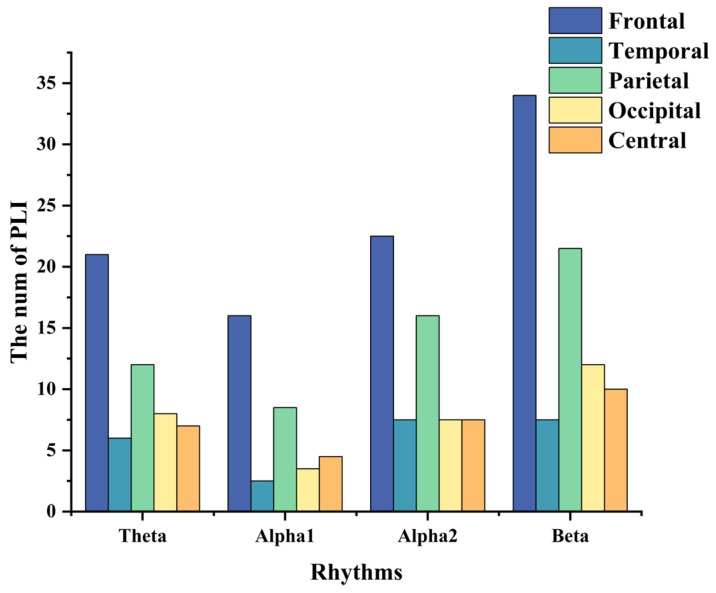
The distribution of key functional connections of theta, alpha1, alpha2, and beta rhythms across various brain regions, including frontal, temporal, parietal, occipital, and central areas. These values are obtained by averaging the key functional connections’ values across the HC, GAD, and DD groups.

Figure 4 shows the feature-selecting results based on the ensemble model. The results show that when the optimal number of feature subsets is 235, the model achieves the best classification performance (accuracy of 97.33%, *F*1-macro score of 97.30%, Gmean-macro of 95.98%, and Kappa value of 97.96%, as shown in Table 5). Figure 5 illustrates the distribution of key functional connections (theta:54, alpha1:35, alpha2:61, and beta:85). Figure 6 provides a statistical overview of the connection strength of key functional connections across different brain regions for theta, alpha1, alpha2, and beta rhythms. The results indicate that the most distinctive functional connections are observed in the beta band among the HC, GAD, and DD groups. Combined with Figure 4, Figure 5 and Figure 6, the results indicate a greater quantity of functional connections in the high-frequency range, especially beta rhythms, demonstrating a significant correlation with the frontal area.

Table 5 displays the results of triple classification based on distinct rhythm-specific key functional connectivity features. The *, **, and *** in the table indicate statistical analyses conducted using a one-way analysis of variance on the output results of the four models (* represents *p* < 0.05, ** represents *p* < 0.01, *** represents *p* < 0.001). Notably, the ensemble model exhibits superior performance in the beta band, achieving an accuracy of 95.20% and an overall accuracy of 97.33% across all key functional connectivity features. This underscores the enhanced discriminative capability of beta band key functional connectivity features in distinguishing among the DD, GAD, and HC groups. The elevated accuracy further emphasizes the effectiveness of ensemble models in diagnosing DD, GAD, and HC.

**Figure 7:**
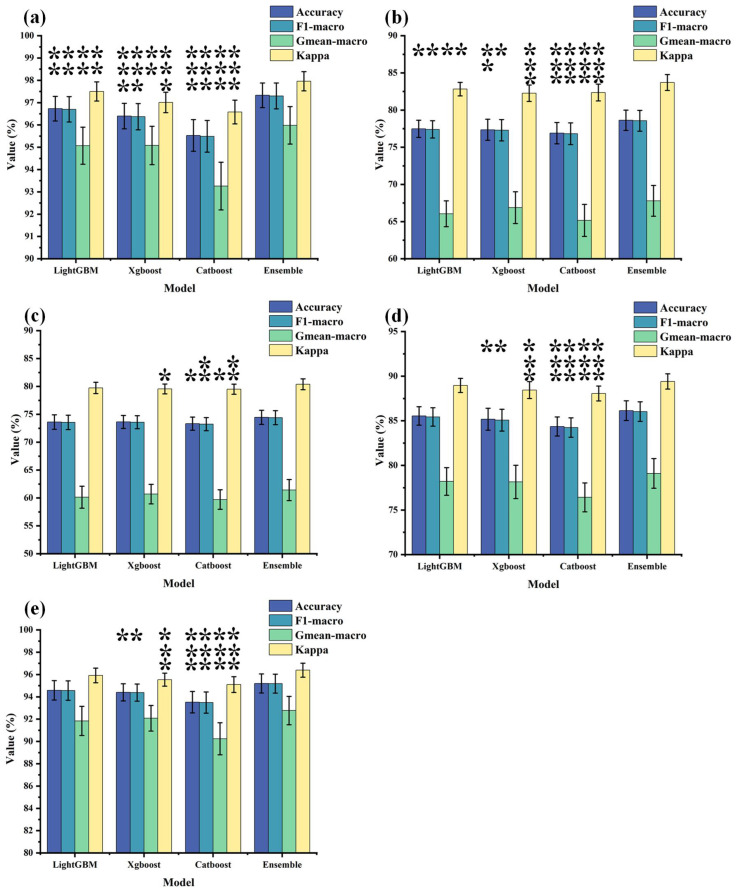
The distribution of output metrics for each model. The bar heights represent the average output results obtained from the optimal feature subsets, with standard deviations also calculated. Furthermore, a post hoc analysis was conducted using multiple comparisons to assess the statistical differences between each sub-model (LightGBM, Catboost, and Xgboost) and the ensemble model, where * represents *p* < 0.05, ** represents *p* < 0.01, and *** represents *p* < 0.001. (**a**) depicts the output results when all rhythmic features are used as inputs. (**b**–**e**), respectively, illustrate the output results when theta, alpha1, alpha2, and beta rhythmic features are used as inputs.

Figure 7 depicts the mean values, standard deviations, and statistical differences of output metrics between each sub-model (LightGBM, Catboost and Xgboost) and the ensemble model. Table 5 outlines the results of the one-way analysis of variance, with Figure 7 illustrating the post hoc analysis using multiple comparisons to determine the statistical differences between the sub-models and the ensemble model. Statistical significance is denoted in the figure, where * represents *p* < 0.05, ** represents *p* < 0.01, and *** represents *p* < 0.001. The ensemble model demonstrates a significant improvement in performance compared to the three individual sub-models, further validating its efficacy for the classification task in this experiment.

## 4. Discussion

This study proposes a diagnostic approach driven by PLI and ML to understand the mechanisms of GAD and DD. In addition, this study also analyzed the impact of different time window feature computations on classification performance. The main conclusions are as follows. Firstly, using a 12 s time window feature calculation under the ensemble model yielded optimal classification performance. Secondly, there was a significant reorganization of brain functional connections in both the DD and GAD groups, with the most prominent alterations identified in the beta frequency band, particularly within the frontal region. Finally, this study innovatively proposes an ensemble learning model based on Autogluon-Tabular, achieving a peak classification accuracy of 97.33%. Notably, the beta frequency band (95.20%) demonstrates the optimal classification performance among the four frequency bands (theta, 78.63%; alpha1, 74.46%; alpha2, 86.14%). This further confirmed the feasibility of using ML to identify DD, GAD, and HC individuals. The following provides a detailed analysis of the obtained results.

### 4.1. Appropriate Time Window Achieves Optimal Classification Performance

Limited research has explored the influence of time windows on the outcomes of identifying DD and GAD, frequently resorting to fixed time window calculations guided by experiential considerations. This study systematically analyzed, for the first time, the impact of feature calculations in different time windows on the classification results of DD and GAD. The results show that a 12 s time window yields optimal classification performance, especially when combined with ensemble model, resulting in a peak accuracy of 97.33%.

The discussion on time windows is more focused on relevant studies on emotion recognition based on EEG [33,34]. Cai et al. [29] indicated that time windows of 4, 5, 6, and 10 s achieve higher accuracy in emotion recognition. Other studies employ fixed time window lengths for feature analysis. For instance, Lin et al. [35] applied a non-overlapping 1 s time window to calculate EEG spectrograms. Yu et al. [36] used a non-overlapping 2 s time window to extract EEG features, and Zhuang et al. treated EEG data in a 5 s time window as materials for empirical mode decomposition [37].

EEG is significantly influenced by emotions, reflected in EEG amplitude–frequency fluctuations [36,38]. Generally, human emotions tend to last for 10 s or more [34]. A longer time window represents less data input, reducing computational costs, while shorter time windows capture transient changes in EEG at the expense of increased computational demands [33]. Choosing an appropriate time window length allows for the optimal utilization of computational resources and time, resulting in improved classification performance. Additionally, we have grounds to infer that the emotional states of individuals with GAD and DD significantly influence the EEG changes. Therefore, a 12 s window reveals greater differences.

Currently, there are no established standards or prior knowledge regarding the time window scale for measuring EEG data. It is known that the calculation of different time window features has a real impact on the results [33], so the discussion of the optimal time window is very important. This study explores the optimal time window length for functional connectivity features based on EEG to enhance the EEG-based identification of DD and GAD. Due to the non-uniformity of our experimental data, the analysis and discussions on time window lengths in this study are specific and need further exploration with a larger dataset to validate these findings.

### 4.2. General Patterns of Brain Reorganization among Three Groups

The functional organization of the brain determines its connectivity [39]. Recent research mentions that mental illnesses result from abnormal brain connectivity, where disruptions or even interruptions in the functional structure of the brain lead to disorders such as DD and GAD [11,17]. Brain functional reorganization is closely associated with abnormalities in brain functional connections [40]. Studies on EEG-related mental disorders often employ analytical methods for functional connections to observe changes in brain reorganization [39,40,41]. In this study, we utilized PLI to compute brain functional connections. By analyzing key functional connections, we aim to understand the pathological mechanisms underlying DD and GAD.

The results indicate a significant reorganization of functional connections across the entire brain, with a notable correlation in the frontal region and beta rhythms for the DD, GAD, and HC groups [21,42]. This demonstrates abnormal functional connections in patients with DD and GAD [11,21]. Research indicates that the brain functional networks in both DD and GAD are deteriorating, with some brain regions undergoing compensatory functional reorganization [41,43]. The prefrontal cortex is particularly affected by DD and GAD compared to other brain regions [44]. The prefrontal cortex is responsible for regulating emotion, decision-making, and memory production [42]. It has been suggested that individuals with DD sacrifice connections between the frontal and parietal regions to achieve long-distance connections [45]. Additionally, the brain functional reorganization in GAD patients involves alterations in both the number [21] and the strength of connections [13]. The abnormal activation in the high-frequency beta band is indicative of anxiety manifestations in both DD and GAD [11,17,21].

In summary, the abnormal connectivity in the frontal cortex can better explain the functional reorganization of the brain in DD and GAD, providing support for future research. However, it should be noted that the experimental paradigms and data used in existing studies vary, making it challenging to conduct consistent comparative analyses. Additionally, the patterns of brain reorganization in individuals with DD and GAD are not entirely consistent [46,47]. There is a need for a standardized experimental paradigm and comprehensive planning to understand the development and progression of changes in brain function and connectivity. Despite these challenges, the current results offer valuable insights into the neural mechanisms underlying DD and GAD.

### 4.3. The Feasibility of Machine Learning for Psychiatric Disorder Diagnosis

ML methods have been widely applied in medical diagnosis and treatment, playing a significant role in addressing mental health disorders [48]. In this study, we innovatively propose a feature selection algorithm aimed at identifying a subset of features that perform well across three base models (LightGBM, XGBoost, and CatBoost), as well as an ensemble model. The results demonstrate that the ensemble model using the optimal feature subset as input achieves a highest accuracy of 97.33%. This surpasses the accuracy obtained without feature selection (96.89%), highlighting the effectiveness of applying ML models to classify DD, GAD, and HC groups, as well as underscoring the crucial role of feature selection in eliminating redundancy. Furthermore, among the four rhythms, the accuracy of the beta rhythm is significantly higher than the others, reaching 95.20% (theta: 78.63%; alpha1: 74.46%; alpha2: 86.14%). This objectively reflects the specificity of the beta rhythm in distinguishing DD, GAD, and HC groups.

While an increasing number of studies focus on diagnostic research for DD and GAD [12], fewer studies are conducted on triple-classification diagnoses with DD, GAD, and HC. More studies tend to analyze two-class scenarios (DD vs. HC, GAD vs. HC). Li et al. utilized feature selection, achieving a classification accuracy of 98.54% ± 0.21% for DD and HC [11]. Their conclusions also highlighted the specificity of the beta band in distinguishing between DD and HC groups. In a binary classification study of GAD and HC, Shen et al. achieved a classification accuracy of 97% [21]. Furthermore, regarding the classification of mental disorders (including DD and AD) and HC groups, Xie et al. employed a combination of EEG and convolutional neural networks (CNN), achieving a classification accuracy of 67.67% [43]. Qi et al. used XGBoost to classify the EEG features of DD and GAD, achieving the highest classification accuracy of 99% [12]. The feature selection algorithm we proposed considers the relative importance of features across multiple training iterations. It is designed to identify a subset of features that demonstrate both stability and significant predictive performance. Through the integration of ensemble learning, our approach contributes to an additional improvement in classification performance. Finally, we achieved the best triple-classification performance, reaching 97.33% accuracy.

However, due to the inconsistency in the experimental paradigm and research methods of this study compared to other referenced studies, specific reasons for the observed differences cannot be determined. It is crucial to establish a standardized experimental paradigm, increase sample size, and develop efficient machine-learning-based methods for processing EEG signals. This is particularly important for the automated assessment of mental disorders such as DD and GAD.

### 4.4. Limitations

The limitations of this study should be acknowledged to provide a comprehensive understanding of its findings and implications, primarily the fact that we included 42 DD patients, 45 GAD patients, and 38 HC adults, which may be considered small and lacking in generalizability. Secondly, the study did not account for the variations in progesterone levels during the menstrual cycle in female participants, which could influence changes in ACEI and EMG signals. Thirdly, the methodology employed in this study involved a relatively simplistic approach using ICA combined with manual artifact removal. Future studies could explore more sophisticated methods for artifact removal, such as ISA, to enhance data quality and minimize potential confounding effects. Lastly, the study utilized fixed EEG frequency bands, which may not fully account for individual variations in frequency band definitions. Future research could benefit from employing individualized frequency band definitions to better capture individual differences in brain activity. In future studies, controlling for factors such as gender, the menstrual cycle and band selection will be prioritized to enhance the robustness and generalizability of the findings.

## 5. Conclusions

This study innovatively proposes a data-driven diagnostic approach for understanding the mechanisms of GAD and DD. The results indicate that a 12 s time window in our experimental paradigm yields superior classification performance, confirming the importance of discussing optimal time windows. Furthermore, brain functional reorganization in the beta band within the frontal region was observed in DD and GAD groups. Lastly, ML serves as an adjunct method for aiding in the diagnosis of mental disorders. In our triple classification study, the ensemble model demonstrated a robust performance at 96.89%, notably improving to 97.33% after feature optimization. This underscores the necessity of feature optimization when employing ML for diagnostic purposes.

## Figures and Tables

**Table 1:** Demographic characteristics of the HC, GAD, and DD.

Features	HC	GAD	DD	*p*
Number	38	45	42	-
Age (year)	38.16 ± 12.36	41.47 ± 9.38	44.55 ± 12.30	0.051
Gender (male/female)	12/26	13/32	10/32	0.74

**Table 2:** Different sample sizes for each group (HC, GAD, and DD) based on varying time window lengths.

Time Window (s)	HC	GAD	DD
2	8506	10,902	9425
4	4163	5371	4632
6	2628	3319	2853
8	1971	2592	2228
10	1530	2048	1747
12	1301	1604	1360
14	880	1122	961
16	873	1077	906

**Table 3:** Optimization variables and ranges of LightGBM, XGBoost, and CatBoost.

Model	Parameter	Description
LightGBM	num_leaves	Uniformint [16,96]
	min_data_in_leaf	Uniformint [2,60]
	feature_fraction	Uniform [0.75,1]
	learning_rate	loguniform [log(5 × 10^−3^),log(0.1)]
XGBoost	depth	Uniformint [3,10]
	min_child_weight	Uniformint [1,5]
	colsample_bytree	Uniform [0.5,1.0]
	learning_rate	Loguniform [log(5 × 10^−3^),log(0.1)]
CatBoost	max_depth	Uniformint [5,8]
	l2_leaf_reg	Uniform [1,5]
	learning_rate	Loguniform [log(5 × 10^−3^),log(0.1)]

**Table 4:** Performance metrics of models segmented at 12 s intervals.

Models	Accuracy	*F*1-Macro	Gmean-Macro	Kappa
Lightgbm	96.44 ± 0.53	96.40 ± 0.50	94.63 ± 0.80	97.26 ± 0.42
Xgboost	95.38 ± 0.53	95.31 ± 0.52	93.04 ± 0.80	96.45 ± 0.41
Catboost	96.72 ± 0.68	96.69 ± 0.70	95.05 ± 1.02	97.50 ± 0.54
Ensemble	**96.89 ± 0.51**	**96.86 ± 0.52**	**95.26 ± 0.77**	**97.65 ± 0.41**

Note: values are mean ± standard deviation; bold indicates highest classification performance.

**Table 5:** Triple classification accuracy results of the LightGBM, XGBoost, CatBoost and ensemble models for saved key PLI features of the DD, GAD and HC groups under different rhythms. The *, **, and *** indicate statistical analyses conducted using one-way analysis of variance on the output results of the four models (* represents *p* < 0.05, ** represents *p* < 0.01, and *** represents *p* < 0.001).

Rhythm	Model	Accuracy	*F*1-Macro	Gmean-Macro	Kappa
All	Lightgbm	96.73 ± 0.55	96.70 ± 0.57	95.07 ± 0.83	97.50 ± 0.43
	Xgboost	96.40 ± 0.57	96.37 ± 0.59	95.08 ± 0.86	97.01 ± 0.46
	Catboost	95.53 ± 0.71	95.49 ± 0.71	93.26 ± 1.07	96.58 ± 0.53
	Ensemble	97.33 ± 0.55 ***	97.30 ± 0.58 ***	95.98 ± 0.84 ***	97.96 ± 0.43 ***
Theta	Ligthgbm	77.48 ± 1.15	77.40 ± 1.16	66.05 ± 1.74	82.82 ± 0.91
	Xgboost	77.35 ± 1.42	77.29 ± 1.43	66.87 ± 2.14	82.27 ± 1.10
	Catboost	76.90 ± 1.43	76.82 ± 1.46	65.16 ± 2.16	82.36 ± 1.12
	Ensemble	78.63 ± 1.37 ***	78.56 ± 1.40 ***	67.79 ± 2.07 ***	83.71 ± 1.07 ***
Alpha1	Ligthgbm	73.62 ± 1.30	73.56 ± 1.29	60.14 ± 1.97	79.74 ± 1.02
	Xgboost	73.64 ± 1.17	73.59 ± 1.16	60.70 ± 1.76	79.55 ± 0.89
	Catboost	73.33 ± 1.17	73.24 ± 1.17	59.72 ± 1.76	79.51 ± 0.90
	Ensemble	74.46 ± 1.26 *	74.40 ± 1.26 *	61.42 ± 1.89 *	80.39 ± 0.97 **
Alpha2	Ligthgbm	85.54 ± 1.03	85.43 ± 1.03	78.20 ± 1.55	88.97 ± 0.79
	Xgboost	85.18 ± 1.23	85.07 ± 1.22	78.15 ± 1.87	88.44 ± 0.95
	Catboost	84.36 ± 1.07	84.24 ± 1.09	76.42 ± 1.62	88.06 ± 0.83
	Ensemble	86.14 ± 1.10 ***	86.03 ± 1.10 ***	79.10 ± 1.66 ***	89.41 ± 0.85 ***
Beta	Ligthgbm	94.58 ± 0.87	94.56 ± 0.87	91.84 ± 1.31	95.92 ± 0.66
	Xgboost	94.40 ± 0.77	94.38 ± 0.77	92.08 ± 1.15	95.54 ± 0.58
	Catboost	93.52 ± 0.96	93.49 ± 0.95	90.24 ± 1.44	95.10 ± 0.71
	Ensemble	95.20 ± 0.85 ***	95.18 ± 0.85 ***	92.77 ± 1.27 ***	96.39 ± 0.63 ***

Note: values are mean ± standard deviation; bold indicates highest classification performance.

## Data Availability

The data presented in this study are available on request from the corresponding author. The data are not publicly available due to privacy and ethical restrictions.
